# Racial residential segregation, socioeconomic disparities, and the White-Black survival gap

**DOI:** 10.1371/journal.pone.0193222

**Published:** 2018-02-23

**Authors:** Ioana Popescu, Erin Duffy, Joshua Mendelsohn, José J. Escarce

**Affiliations:** 1 Division of General Internal Medicine and Health Services Research, David Geffen School of Medicine, Los Angeles, CA, United States of America; 2 The RAND Corporation, Santa Monica, CA, United States of America; West Chester University of Pennsylvania, UNITED STATES

## Abstract

**Objective:**

To evaluate the association between racial residential segregation, a prominent manifestation of systemic racism, and the White-Black survival gap in a contemporary cohort of adults, and to assess the extent to which socioeconomic inequality explains this association.

**Design:**

This was a cross sectional study of White and Black men and women aged 35–75 living in 102 large US Core Based Statistical Areas. The main outcome was the White-Black survival gap. We used 2009–2013 CDC mortality data for Black and White men and women to calculate age-, sex- and race adjusted White and Black mortality rates. We measured segregation using the Dissimilarity index, obtained from the Manhattan Institute. We used the 2009–2013 American Community Survey to define indicators of socioeconomic inequality. We estimated the CBSA-level White–Black gap in probability of survival using sequential linear regression models accounting for the CBSA dissimilarity index and race-specific socioeconomic indicators.

**Results:**

Black men and women had a 14% and 9% lower probability of survival from age 35 to 75 than their white counterparts. Residential segregation was strongly associated with the survival gap, and this relationship was partly, but not fully, explained by socioeconomic inequality. At the lowest observed level of segregation, and with the Black socioeconomic status (SES) assumed to be at the White SES level scenario, the survival gap is essentially eliminated.

**Conclusion:**

White-Black differences in survival remain wide notwithstanding public health efforts to improve life expectancy and initiatives to reduce health disparities. Eliminating racial residential segregation and bringing Black socioeconomic status (SES) to White SES levels would eliminate the White-Black survival gap.

## Introduction

Despite substantial improvements in US life expectancy, White-Black disparities in survival remain wide.[1–4] The pathways underlying these disparities are not fully understood, but social determinants of health are deemed to play a primary role.[5–8] Low socioeconomic status (SES), which is more prevalent among black populations, is linked to a broad range of adverse health outcomes including higher burden of chronic disease,[9] worse self-reported health status,[10] and higher mortality rates.[11] Many factors have been invoked to explain SES disparities, but systemic racism and its prominent manifestation, racial residential segregation, are considered key in the perpetuation of White-Black inequality.[12]

Residential segregation refers to the spatial separation of population groups along racial or ethnic lines. Patterns of residential segregation have been historically shaped by systemic interpersonal and institutional racism. Prior to the 1960s, racial residential segregation was both legal and ubiquitous in the United States, enforced by social and public policies such as the Jim Crow laws in the South and the Federal Housing Administration’s early lending (red-lining) policies.[13] With the passage of the Civil Rights Act in 1964 and of the Fair Housing Act (FHA) in 1968 segregation officially ended. Nonetheless, de facto segregation persists through a variety of political and social mechanisms.[14] As a consequence, Blacks currently live under a level of segregation higher than that of any other racial/ ethnic group.[15]

**Fig 1:**
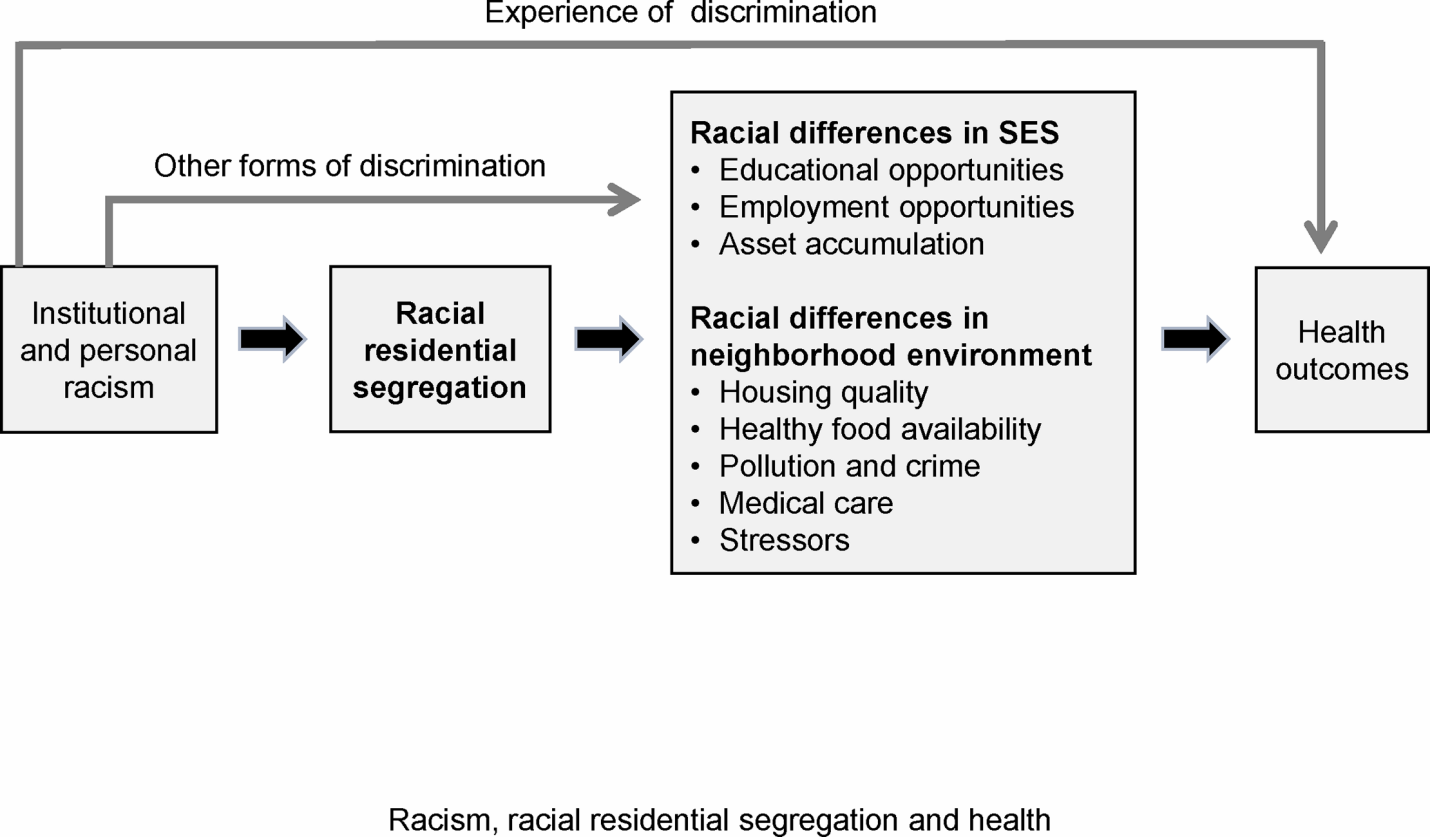
Racism, racial residential segregation and health.

The theoretical framework for the current study is based on the concept that social factors are fundamental causes of inequalities in health.[6] Because of its pervasive effects on everyday life and on the social and built environment, racial residential segregation (henceforth segregation) is widely seen as a primary cause of racial disparities in health and survival.[5, 16–18] Segregation affects health through multiple pathways. First, segregation shapes racial differences in SES. Black neighborhoods in highly segregated metropolitan areas tend to have limited access to educational and labor market opportunities, which contribute to racial income inequality, high levels of unemployment and concentrated poverty.[5, 8, 19] Second, segregation influences health through its negative effects on neighborhood conditions, including crowded housing, poor housing quality, and lack of amenities such as recreational facilities and establishments offering healthy food choices.[5, 16] Black neighborhoods in highly segregated metropolitan areas may receive fewer and lower quality services from local municipalities and the pervasive disadvantages may contribute to social disorganization and family disruption, diminished trust among members, and increased crime.[5] Lastly, segregation has negative effects on health care delivery for black individuals, including lower rates of health insurance (often tied to lower employment rates), and diminished access to high quality providers and treatments.[20, 21] The pathways through which racial residential segregation and other forms of systemic racism lead to, and ultimately to health inequalities are summarized in Fig 1.

The objective of this study was to evaluate the relationship between racial residential segregation and the White-Black gap in survival, and the extent to which socioeconomic inequality explains this relationship. Our study contributes to the field in several ways. We employed contemporary Census data to ensure that our findings reflect the current state of segregation and survival disparities; we used race-specific socioeconomic indicators to account for the fact that the racial gap in SES may differ across CBSAs; and we estimated separate models for men and women (to assess whether effect size differs by gender). Additionally, the study outcome was the gap in the probability of survival from age 35 to 75. This metric is intuitively easy to understand and complements earlier findings using mortality rates. Lastly, we used the results of our analyses to predict how large the survival gap would be if segregation were eliminated and the socioeconomic status of Blacks could be brought to the level of White socioeconomic status.

Given the rising White-Black income gap during and after the recent recession,[22] and the persistent high levels of White-Black residential segregation, understanding how these factors interplay in the current historical and socioeconomic context and how they influence health is of particular public and health policy interest.

## Methods

### Data sources

The primary data source for the study was the compressed mortality file for 2009–2013, obtained from the Centers for Disease Control and Prevention (CDC) Wide-Ranging Online Data for Epidemiological Research (WONDER) system, a publicly available database.[23] CDC WONDER provides county-level, race- and gender-specific data on death and population counts by 5- and 10-year age groups. We aggregated these data at the Core Based Statistical Area (CBSA) level. CBSAs collectively represent both metropolitan and micropolitan areas in the United States. We used CDC WONDER data to create our study cohorts and to compute age/gender/race specific mortality rates. We obtained a CBSA-level measure of segregation Manhattan Institute,[24] and measures of SES from the American Community Survey for 2009–2013.

### Study sample

The study sample consisted of Black and non-Hispanic White (henceforth White) men and women aged 35–75 captured in the CDC WONDER database, aggregated at the CBSA level. To protect against the potential disclosure of personal health information, CDC WONDER suppresses data for any gender/race/age group with fewer than 10 deaths in a county. Thus, we imputed death counts for gender/race/age groups with missing data using a previously published algorithm that pools mortality rates for corresponding groups from other counties within a larger metro area to estimate the missing counts.[25]

We restricted our study sample to 102 CBSAs that met two criteria: total Black population >40,000 and imputation of deaths required for less than 10% of the Black population. Across these CBSAs, we only needed to impute deaths for 2.9% of the Black population. Moreover, because the groups that were missing mortality data were young and had low death rates, we estimate that we only imputed 0.5% of deaths. To evaluate the robustness of our results, we performed sensitivity analyses using CBSAs where imputation of deaths was required for less than 5% (N = 67) and for less than 15% (N = 121) of the Black population.

### Study variables

The outcome variable for this study was the White-Black gap in the probability of survival from age 35 to 75, computed for the entire sample and separately for men and women, using standard life table techniques.[26] Death and population counts were aggregated at the CBSA level. We elected to measure mortality beginning at age 35 to reduce the impact of violent causes of death, which are more prevalent among younger individuals, and enable the outcome to capture mainly the chronic effects of segregation and SES on death due to illnesses.[27] (According to CDC data, the percentage of deaths due to unintentional injuries/homicide/suicide out of all deaths in the 35–74 age group is 11% for white men, 8% for black men, 7% for white women and 4% for black women.)

The key independent variable for the study was racial residential segregation. We measured racial residential segregation at the CBSA level using the White-Black dissimilarity index, which has been widely employed in studies of segregation and health. The dissimilarity index, which ranges from 0 to 1, is a measure of evenness and assesses the degree to which the proportions of minority and majority group members within individual census tracts are similar to the proportions in the CBSA. We selected dissimilarity, a measure of evenness, to quantify the degree of residential segregation at the CBSA level because we believe it is conceptually the most appropriate for our study. Evenness refers to the differential distribution of black and white individuals across census tracts within the CBSAs, that is, to their spatial separation. High levels of dissimilarity signify that blacks and whites have little common area of residence within the CBSA, and, the more spatially separated blacks and whites are within a CBSA, the more likely they are to lead separate lives in neighborhoods increasingly different in quality and in access to influence and resources. In keeping with the study framework (Fig 1), racial residential segregation, as measured by dissimilarity, affects health outcomes through racial differences in socioeconomic conditions, neighborhood quality, and access to health care.

Other explanatory variables included the proportion of black individuals in each CBSA and measures of SES. We measured SES using five variables from the 2009–2013 ACS, obtained separately for Black and White men and women: median household income, percent population below the poverty line, percent population aged 25 or older with less than a high school diploma, percent males aged 16 or older who are unemployed, and percent households with children who have a female head-of-household. All the selected component variables have been previously included in published SES indices.[28–31] Four variables are common measures of SES (income, poverty, education and employment); a fifth variable, the percent of households with children headed by women, was included as a measure of social disorganization and family disruption,[32, 33] which represent another potential pathway for segregation effects on health. For each CBSA we computed a race-specific SES index following a previously published method.[28] First, we standardized each individual variable in each CBSA, using the race-specific mean and standard deviation across CBSAs to calculate z-scores for each CBSA. We then added the z-scores for the five variables to create the race- and gender-specific SES index. Cronbach’s alpha (a measure of internal consistency) was 0.78 for the White SES index and 0.81 for the Black SES index.

### Statistical analysis

We used linear regression models to evaluate the association between segregation and the racial gap in the probability of survival from age 35 to 75 for the Black and White population overall and for men and women separately. We estimated the following model:

$$Y_{w-b,c}=\alpha +\beta D_{c}+\gamma P_{c}+\theta_{w}X_{w,c}+\theta_{b}X_{b,c}$$

where *Y*_*w−b,c*_ is the White-Black gap in the probability of survival from age 35 to 75 in CBSA *c*; *D*_*c*_ is the dissimilarity index in CBSA *c*; *P*_*c*_ is the percent of the population black in CBSA *c*; *X*_*r*,*c*_ (*r = w*, *b*) represents the SES measure (i.e., income and SES index, in the respective models) for whites and blacks in CBSA *c*; and the Greek letters are regression coefficients to be estimated. Of note, β represents the change in the White-Black survival gap for an increase of one unit in the dissimilarity index.

We estimated three sequential regression models to elucidate the association between survival and segregation and the degree to which this association was explained by socioeconomic factors. Thus in Model 1, the base model, the explanatory variables were segregation (the dissimilarity index) and the proportion of black residents in the CBSA, which is considered an important control variable that should be included in analyses of the association between segregation and health status.[27] In Model 2 we added the race-specific median household income in the CBSA. Finally, in Model 3 we replaced median household income with the race-specific SES index in the CBSA (described earlier), a broader and more comprehensive measure of socioeconomic status. We used the sandwich estimator to correct the standard errors of the regression coefficients for heteroskedascity.

To make the regression results easier to interpret, we used the estimated coefficients from each model to predict the White-Black gap in the probability of survival from age 35 to 75 for CBSAs with the lowest (0.23) and highest (0.78) observed dissimilarity indexes. The percent of the black population was set to its mean and the race-specific socioeconomic characteristics were set to the means for the White population in the simulations, which has the effect of bringing the socioeconomic status of Blacks up to the level of Whites in the predictions. Our intent was to isolate the effect of segregation on the survival gap equalizing the socioeconomic status of Blacks and Whites.

## Results

### Descriptive data

Black men and women in the 102 study CBSAs were less likely than their White counterparts to survive from age 35 to 75 (Table 1). White women were the most likely to survive to 75 years (75%), followed by Black women (66%), White men (63%), and Black men (49%). The survival gap was 14% for men and 9% for women. Across all CBSAs, measures of socioeconomic status were significantly worse for Blacks as compared to Whites (Table 1). The mean dissimilarity index for the study CBSAs was 0.51 (SD, 0.12), and the mean proportion of black residents was 20% (SD, 12%).

**Table 1:** Survival from age 35 to 75 and socioeconomic characteristics of White and Black men and women within 102 US CBSAs.

	White population Mean (SD)	Black population Mean (SD)
**Survival**		
Percent surviving to age 75, overall	69.4 (4.3)	58.2 (4.5)
Percent surviving to age 75, men only	62.9 (5.1)	49.3 (5.6)
Percent surviving to age 75, women only	74.9 (3.5)	65.9 (3.8)
**Socioeconomic characteristics**		
Median household income ($1,000s)	60.2 (12.4)	34.1 (7.7)
Percent living in poverty	10.3 (2.6)	28.8 (5.7)
Percent with less than high school diploma	9.0 (2.7)	17.7 (4.5)
Percent unemployed, males >16	7.3 (1.7)	13.0 (2.5)
Percent female head-of-household	9.3 (1.2)	29.8 (3.1)
SES Index	7.0 (2.8)	-13.3 (4.4)

**Fig 2:**
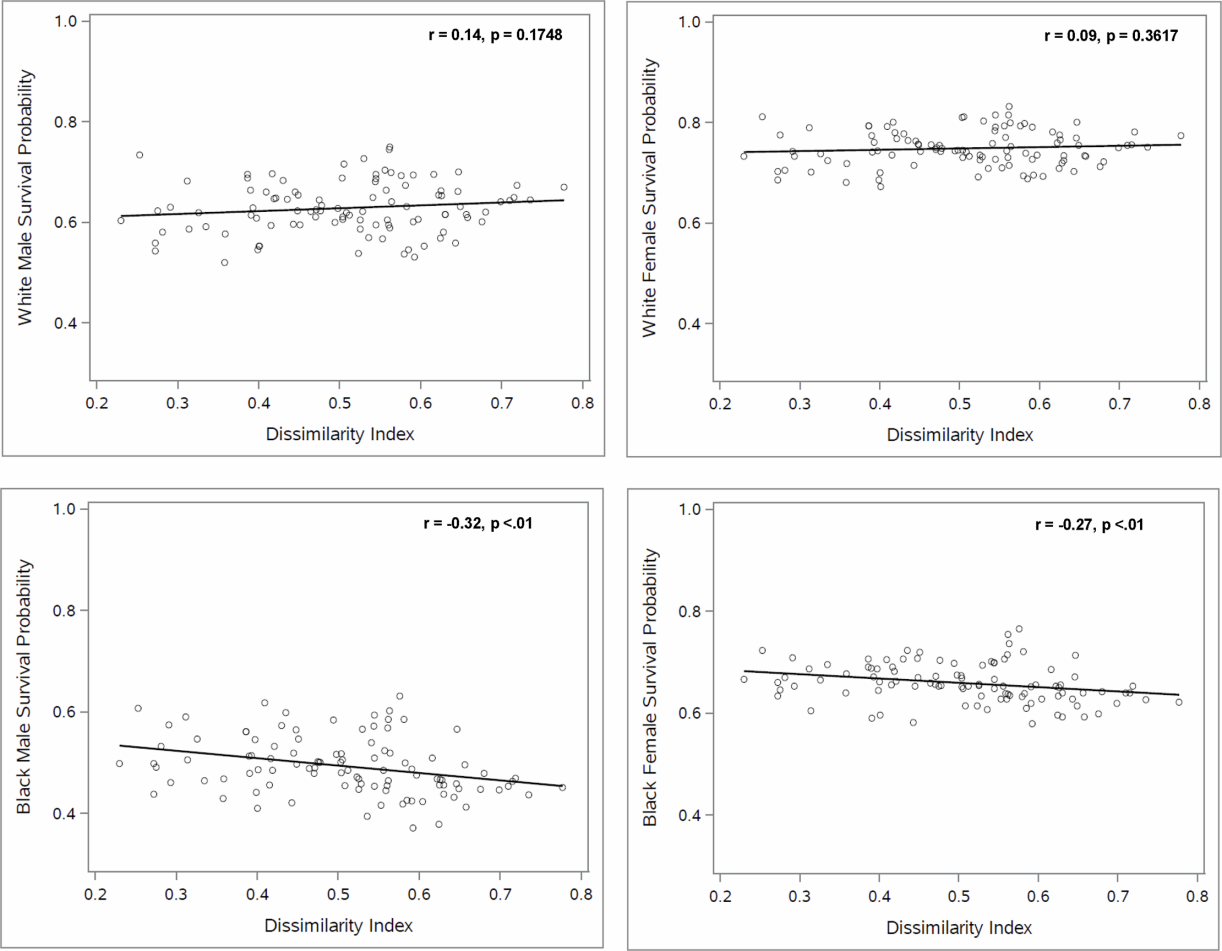
The relationship between racial residential segregation and the probability of survival for Black and White individuals from 35 to 75.

**Fig 3:**
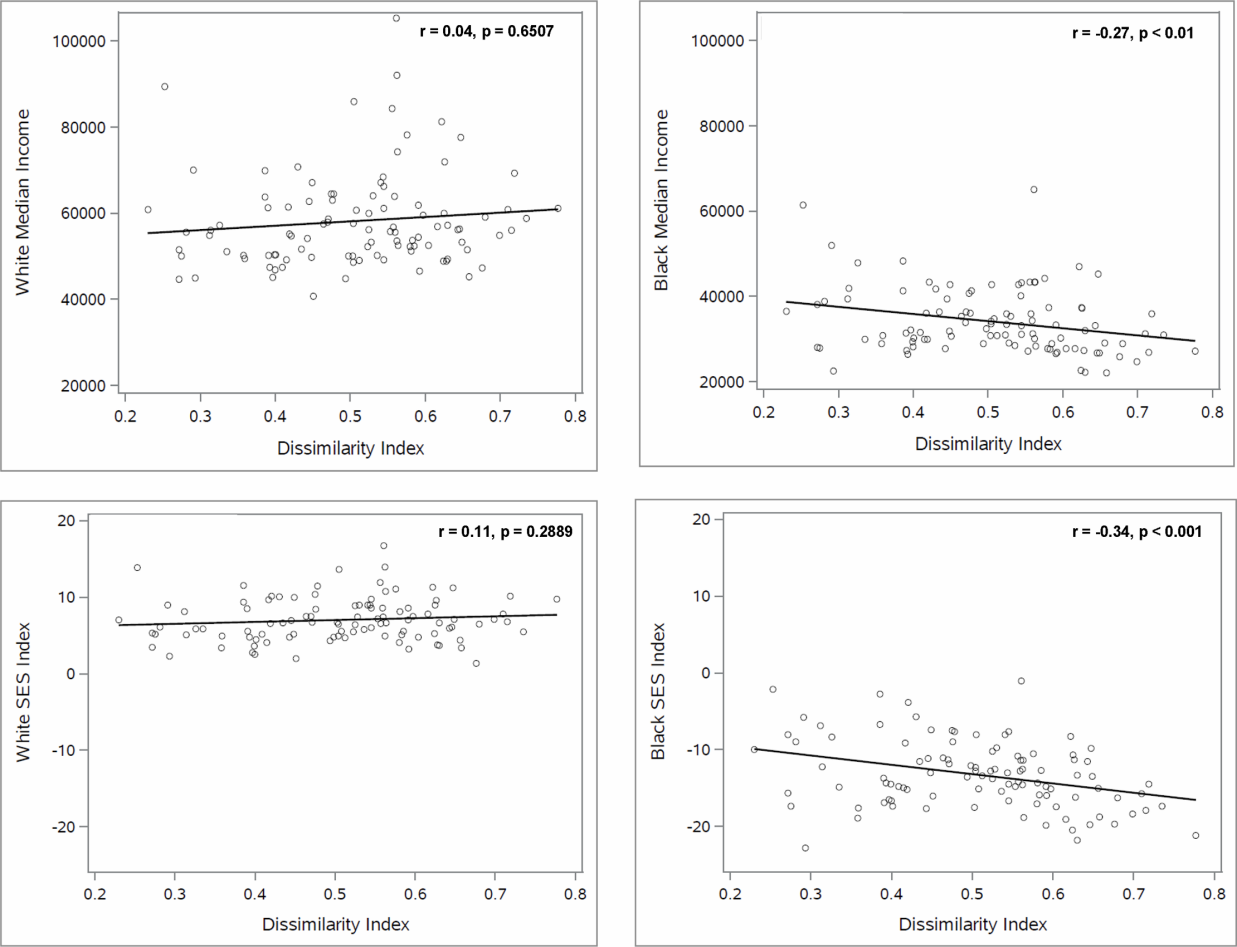
The relationship between racial residential segregation, median household income and SES index for Black and White individuals aged 35–75.

**Fig 4:**
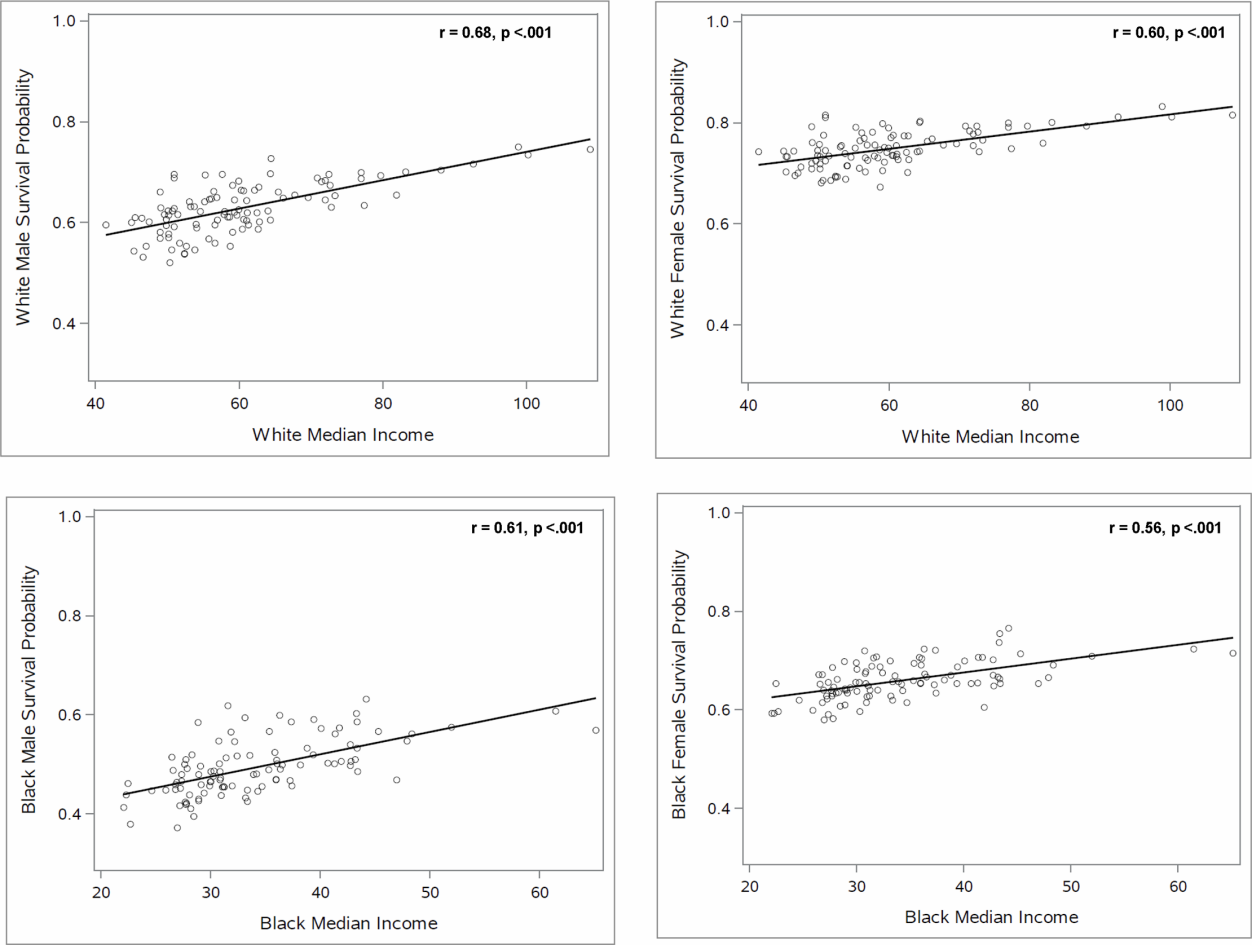
The relationship between median household income and the probability of survival for Black and White individuals from 35 to 75.

**Fig 5:**
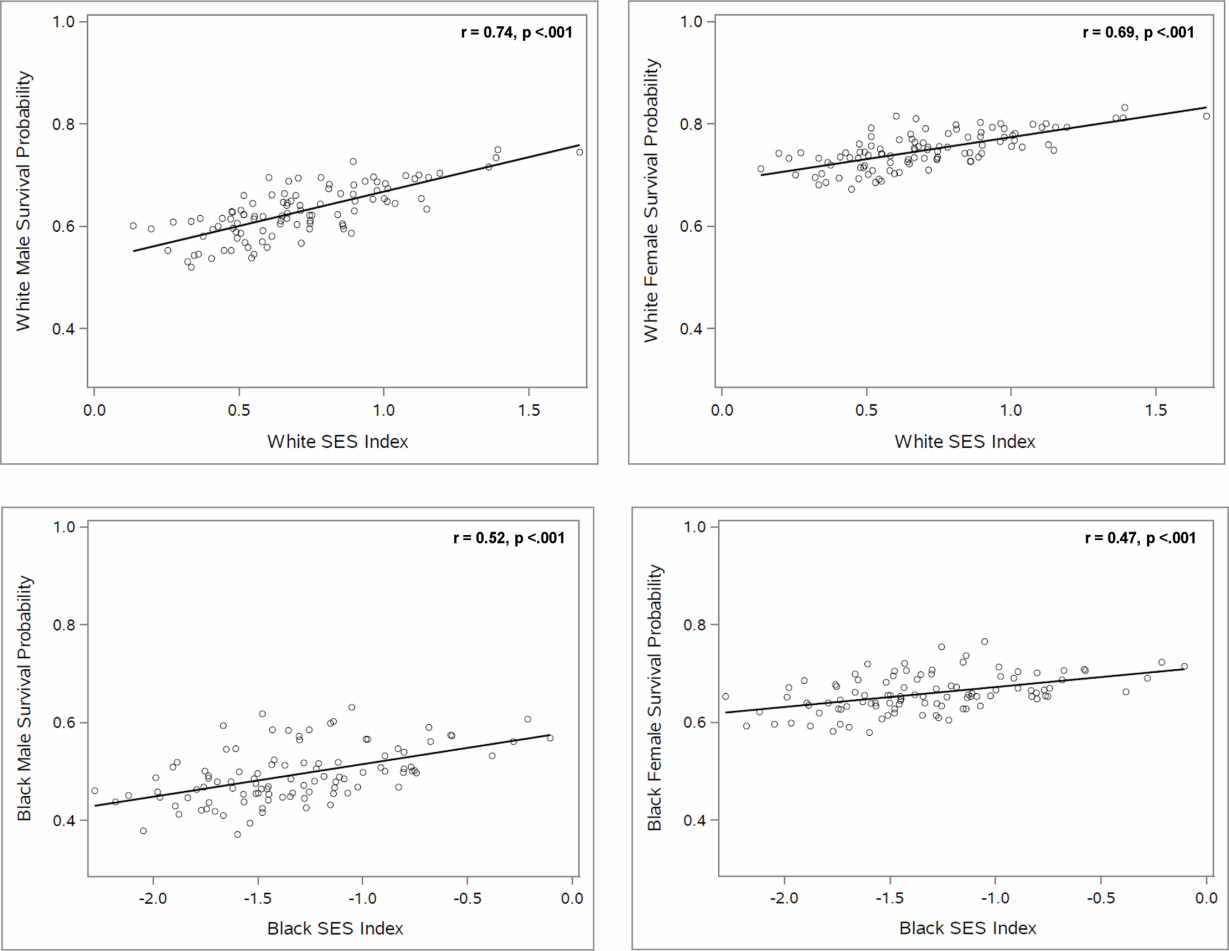
The relationship between SES index and the probability of survival for Black and White individuals from 35 to 75.

As shown in the scatterplots in Fig 2, the association between the probability of survival from age 35 to 75 and racial residential segregation differed strikingly across the two racial groups. Specifically, whereas the probability of survival was uncorrelated with the dissimilarity index for White men and women, survival was negatively associated with the measure of segregation, dissimilarity, for Black men and women (r = -0.32, p < .01 and r = -0.27, p < .01, respectively). Similarly, the association between median household income and segregation, and between the SES index and segregation (Fig 3) differed by race. Thus, whereas median household income and SES index were uncorrelated with the dissimilarity index for Whites, Black median household income and SES index were negatively correlated with dissimilarity (r = -0.27, P < .01, and r = -0.34, p < .001 respectively). By contrast, the association between the probability of survival from age 35 to 75 and median household income or SES index was similar for Whites and Blacks (Figs 4 and 5), with both races (and both genders) having higher survival in CBSAs where they had higher median household income and SES index.

The findings in Figs 2–5 indicate that the White-Black survival gap is greater in more segregated than in less segregated CBSAs and suggest that a portion of this association may be explained by the association between the White-Black difference in median household income (or SES index) and segregation. Our regression analyses shed light on this question.

### Regression results

Table 2 presents the results from Models 1–3, described earlier, that assessed the association between the White-Black survival gap and segregation and sequentially adjusted for the proportion of Blacks in the CBSA, median household income, and the SES index.

**Table 2:** The contribution of residential segregation, as measured by dissimilarity (D), to the white-black survival gap from age 35 to 75 overall, and for men and women separately.

	White-Black Survival Gap					
	Overall		Men		Women	
	Point estimate	95% CI	Point estimate	95% CI	Point estimate	95% CI
**Model 1**^**†**^						
**D = 0.23**	6.5%	1.7–11.4%	7.5%	1.4–13.5%	6.1%	1.6–10.6%
**D = 0.78**	14.9%	7.3–22.4%	19.3%	10.0–28.6%	11.8%	4.9–18.8%
Change in Black-White survival gap for a change in D from 0.23 to 0.78	8.4%***		11.8%***		5.7%***	
Adjusted R-squared	0.27		0.32		0.20	
**Model 2**^**‡**^						
**D = 0.23**	8.6%	-8.0–25.3%	10.2%	-10.0–30.4%	7.8%	-8.5–24.0%
**D = 0.78**	12.7%	-6.8–32.2%	16.6%	-7.1–40.3%	10.1%	-8.9–29.1%
Change in Black-White survival gap for a change in D from 0.23 to 0.78	4.1%**		6.4%***		2.3%	
Adjusted R-squared	0.43		0.50		0.31	
**Model 3**^**¥**^						
**D = 0.23**	8.3%	3.1–17.3%	9.6%	3.3–15.8%	7.5%	2.5–12.4%
**D = 0.78**	12.9%	4.8–24.9%	17.0%	7.1–26.9%	10.2%	2.5–18.0%
Change in Black-White survival gap for a change in D from 0.23 to 0.78	4.6%***		7.4%***		2.7%	
Adjusted R-squared	0.40		0.46		0.30	

**p < .01

***p < .001

^**†**^Model 1 accounts for CBSA-level dissimilarity and proportion of black residents

^**‡**^Model 2 accounts for CBSA-level dissimilarity, proportion of black residents and race-specific median household income

^**¥**^Model 3 accounts for CBSA-level dissimilarity, proportion of black residents and race-specific SES index

In analyses accounting only for segregation (Model 1), a change in dissimilarity from 0.78 (highest segregation) to 0.23 (lowest segregation) was associated with a decrease in the white black survival gap of 8 percentage points for Blacks overall, 11 percentage points for Black men and 6 percentage points for Black women (p < .001).

Adding race-specific median household income (Model 2) or the SES index (Model 3), centered on the White population means, partially explained the effect of segregation on Black survival. Under the lowest segregation scenario, and with Black SES assumed to be at the White SES level scenario, the White- Black gap in predicted probabilities of survival was eliminated.

Sensitivity analyses using different cutoffs for the proportion of data imputed (i.e., 5% and 15%) showed similar results.

## Discussion

In a contemporary national sample of Black and White individuals living in major US CBSAs, we found that Blacks had a lower probability of survival from age 35 to 75 than their White counterparts. Racial residential segregation was negatively associated with Black survival but did not affect survival for Whites. Consequently, the White-Black survival gap was substantially greater in more segregated compared with less segregated CBSAs. Additionally, we found that the association between segregation and the White-Black survival gap was partly, but not fully, explained by racial differences in socioeconomic status. The persistence and magnitude of current White-Black health disparities should be of major policy concern.

Our results concur with prior research showing that residential segregation is associated with higher mortality for Blacks, and that the effects of segregation are explained to various degrees by SES.[4, 34–40] As compared to other studies using death rates as a primary outcome, our outcome, the probability of survival from age 35 to 75, focuses on the age range in which the influence of socioeconomic inequality and racism on health is most prominent and is likely to operate mainly through illness rates and severity. (The relationship between socioeconomic status and racial differences in mortality is greatly attenuated at older ages.[37]). Further, most prior studies that controlled for SES in analyses of segregation and health used a single measure of SES for the entire population. Only a couple of studies[36, 40] used race-specific SES measures, which is necessary to account for the fact that Blacks and Whites within the same metro area may live under very different socioeconomic conditions. By employing race-specific CBSA-level measures of socioeconomic status, our study was able to show that segregation specifically influences Black socioeconomic status and survival.

Our analyses also reveal that the effects of segregation on the White-Black survival gap are only in part explained by socioeconomic factors. Racial differences in living conditions fostered by segregation, including differences in housing and environmental quality, the social and built environment, the availability and quality of medical services, and pervasive interpersonal racism and discrimination may all contribute to worse Black health.

Importantly, our study suggests that racial residential segregation takes a particularly high toll on Black men. Only a handful of prior studies have examined the effects of segregation separately for men and women;[4, 36, 41, 42] two of these studies evaluated an isolated metro area, and most relied on data from the 1990s. There are multiple reasons why segregation may have larger effects on the health of adult Black men. First, men may be subjected to higher levels of institutional discrimination and pervasive negative stereotypes in the labor market. Prior research has shown that perceptions of racism and discrimination in the labor market may be higher among Black men than women.[43] Perceived discrimination in turn has detrimental effects on health.[19] Incarceration, which has tremendous negative effects on individual, family and community well-being, is yet another form of institutional discrimination that disproportionately affects black men.[44] Finally, the recent economic downturn has disproportionately affected Blacks. While pre-recession data showed that the White-Black income and wealth gap were slowly declining, post-recession analyses show a significant widening of the racial gap in income and wealth, and much higher rates of unemployment for Black compared to White men.[45]

Our study underscores that White-Black disparities in survival remain sizeable, driven by residential segregation and socioeconomic inequality. The solutions proposed have been many and varied, but what they have in common is a focus on ameliorating the differences in living conditions between black and white neighborhoods in highly segregated metropolitan areas.[46] Additionally, federal and state policymakers are expanding initiatives targeted at better employment opportunities and pathways to higher education for minority students.[47]

Our study has several limitations. First, the study was an ecological analysis of the association between the White-Black survival gap and contemporaneous measures of residential segregation and socioeconomic status. The effects of segregation and socioeconomic conditions on health are likely to reflect exposures that cumulate over the life course and may change over time as CBSAs change and people move. To the extent contemporaneous measures fail to capture accurately these cumulative exposures, they could be considered to measure the cumulative exposures with error. A standard statistical result suggests that, as a consequence, our analyses may understate the associations between the independent variables and survival.[48] Second, we evaluated the average effects of racial residential segregation and socioeconomic conditions on survival in different CBSAs. Individual characteristics and conditions in particular neighborhoods are likely to affect health as well, but we did not have data on these factors. Third, our study does not explore the specific mechanisms through which segregation and socioeconomic status may affect survival. Nonetheless, the ecological approach has proved extremely powerful in identifying relationships between the social conditions in an area and health outcomes. Our study is in this tradition.

The findings of this study provide evidence to support the notion that attempts to improve specific aspects of living conditions in disadvantaged Black neighborhoods, while imperative, are unlikely ever to be able to make a big enough difference. A direct attack on racial residential segregation, the root cause of differences between Whites and Blacks in living conditions and socioeconomic opportunity,[5] is also needed to reduce and ultimately eliminate White-Black health disparities.

The passing of the FHA in 1968 officially prohibited the discrimination concerning the sale or rental of housing based on race. However, the FHA also required the government to actively break down segregation and promote residential integration (i.e., to "affirmatively further fair housing"), a mandate that has been largely forgotten or ignored. Examples of FHA violations have been extensively documented,[49] including restrictive zoning laws that limit the number and size of multi-family housing or the concentration of new affordable housing in undesirable neighborhoods to name a few. In some instances, irregularities have continued even in the face of court rulings.[50] Other federal programs (e.g., the HOPE program) that focus on improving conditions in segregated communities (e.g., by demolishing distressed projects and replacing them with mixed-income housing within the same community) are promoting the equally unjust and untenable “separate but equal” doctrine.

Despite the general inertia, some local governments have shown that integration is possible. In the years immediately following the enactment of the FHA, Montgomery County in Maryland, one of the wealthiest counties in the US, passed a law enforcing the availability affordable public housing units and integrated housing development for lower-income residents. During the following decades, the county’s black population more than tripled, while segregation is well below average and Montgomery County remains one of the nation's wealthiest counties.

Several public and private programs are now in place in major metropolitan areas to help promote fair housing policies, and may serve as positive examples and starting points for integration efforts. These programs assist minorities in finding and securing affordable rental housing, expand the availability of mortgage credits for low-income households, provide housing quality inspections and even offer post-move counseling to help minorities get access to areas of better socioeconomic opportunity.[51] Nevertheless, as our study shows, no initiative that merely focuses on the socioeconomic consequences of segregation without fully committing to tackle the root cause itself is likely to succeed.
